# Pre-radiofrequency ablation MRI imaging features predict the local tumor progression in hepatocellular carcinoma

**DOI:** 10.1097/MD.0000000000023924

**Published:** 2020-12-24

**Authors:** Zhouchao Hu, Nannan Yu, Heping Wang, Shibo Li, Jingang Yan, Guoqiang Zhang

**Affiliations:** a Interventional Diagnosis and Treatment Center; b Department of Anesthesiology; c Department of Radiology; d Department of Infection Disease; e Department of Hepatobiliary Surgery, Zhoushan hospital of Zhejiang University, No.739 Dingshen road, Zhoushan city, Zhejiang province, China.

**Keywords:** local tumor progression, magnetic resonance imaging, nomogram, radiofreqeuncy ablation

## Abstract

To investigate whether MRI features could preoperatively predict local tumor progression (LTP) in patients with hepatocellular carcinoma (HCC) treated with radiofrequency ablation (RFA) as the first-line treatment and improve a novel predictive model through developing a nomogram including various conventional MRI parameters. 105 patients with HCCs who had received RFA were enrolled. All patients had undergone conventional MRI before RFA. Uni- and multivariable analyses for LTP were assessing using a Cox proportional hazards model. The developed MRI-based nomogram was further designed based on multivariable logistic analysis in our study and the usefulness of the developed model was validated according to calibration curves and the C-index. Rim enhancement (hazard ratio: 2.689, *P* = .044) and the apparent diffusion coefficient (ADC) values (hazard ratio: 0.055, *P* = .038) were statistically significant independent predictors of LTP after RFA at multivariable analysis. The performance of the nomogram incorporating two MRI parameters (with a C-index of 0.782) was improved compared with that based on rim enhancement and ADC alone (with C-index values of 0.630 and 0.728, respectively). The calibration curve of the MRI-based nomogram showed good conformance between evaluation and observation at 0.5, 1, and 1.5 years after RFA. The preliminary predictive model based on MRI findings including rim enhancement and ADC value could be used preoperatively to estimate the risk of LTP of HCC after RFA as the first-line treatment.

## 1 Introduction

Hepatocellular carcinoma (HCC) is the second leading cause of cancer-related deaths among people in China. For early-stage HCCs, surgical resection and radiofrequency ablation have been the curative therapies. Several clinical trials reported that radiofrequency ablation (RFA) can be as safe and effective as hepatic resection for HCC.^[1]^ Furthermore, RFA is faster and simpler to operate and less invasive requires shorter hospitalization for patients. Considering these factors, RFA has been suggested as the first-line treatment option for HCC in several medical institutions. A previous study reported that the 5-year overall survive rate of patients with HCCs who undergo RFA is 60%.^[2]^ Despite the advantages of the therapy for HCC, several studies have reported the risk of local tumor progression (LTP) of HCC treated with radiofrequency ablation (RFA) is still high with tumor recurrence developing in 20% of patients at 5 years.^[3]^ Therefore, it is of great importance to identify preoperative predictors for prognosis of HCC patients treated with RFA because these factors may contribute to making decisions about the different treatments for HCC. Several postoperative biomedical parameters, such as pathological results and microRNA expression were considered as important predictors to assess to prognosis of HCC treated by RFA.^[4,5]^ However, to date, noninvasive and accurate factors of prognosis are still not established. Although several clinical predictors of prognosis of HCC treated with RFA as first-line therapy, such as ChildPugh class, alphafetoprotein (AFP) level and age have been reported, there is no consensus.^[6,7]^ Recently, several preoperative imaging features of HCC have been used to predict early recurrence of HCC following hepatic resection, but preoperative radiological features are rarely used to predict the prognosis of HCC treated with RFA. Although Kang et al have used MRI with gadoxetic acid to predict LTP after RFA in patients with HCC, the lack of popularity of this contrast agent limits its application.^[8]^ Moreover, to our knowledge, the combination of radiological parameters for predicting postoperative LTP has not been studied.

Therefore, the purpose of our study was to evaluate the potential role of conventional preoperative MR features in the prediction of LTP in HCC patients after RFA as first-line treatment. Additionally, we tried to combine various imaging parameters and develop a more clinically applicable model to improve the accuracy and efficiency of prediction in the prognosis of HCC.

## 2 Methods

### 2.1 Patient selection

**Figure 1:**
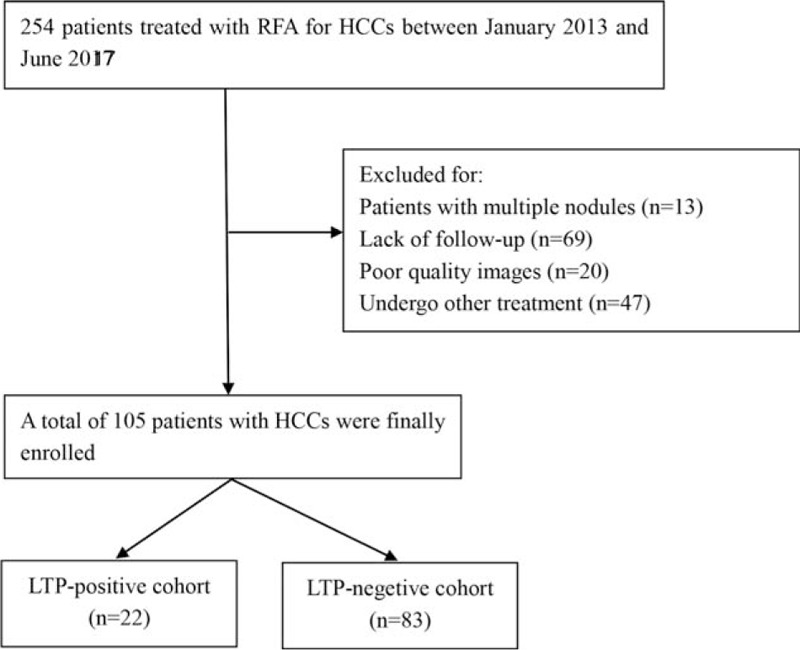
Flow chart of patients with HCC selection. RFA, radiofrequency ablation; HCC, hepatocellular carcinoma.

This retrospective study was approved by the institutional review board of our institution and the requirement for informed consent was waived (Ethic approve ID: B2016-071). Between January 2013 and June 2017, 254 consecutive patients were treated with RFA. The inclusion criteria of the enrolled patients were as follows (Fig. 1):

(1) recent diagnosis of HCC without synchronous extrahepatic metastases confirmed by the typical dynamic pattern of the tumor on contrast-enhanced CT/MRI and/or biopsy;
(2) a largest diameter of 5 cm;
(3) less than 3 nodules without portal vein thrombosis or extrahepatic metastases;
(4) platelet count and no less than 50 × 10^9^/mm^3^ and prothrombin activity no less than 50%;
(5) patients who underwent preoperative contrast-enhanced MR examinations, including diffusion-weighted imaging performed within 10 days prior to RFA and
(6) patients who did not undergo other treatment prior to surgery and have no history of extrahepatic cancer.

149 patients were excluded for the following:

(1) Lack of MR imaging data/poor-quality MR images (n = 20);
(2) patients with multiple nodules (n = 13);
(3) patients with a short-term follow-up, less than 3 months (n = 69); and
(4) patients with other treatment prior to RFA (n = 47).

First-line therapy was considered as no prior treatments for patients with HCCs at the time of the initial diagnosis, as previous reported. Finally, 105 patients with HCCs treated with RFA were enrolled in our study.

### 2.2 MRI acquisition

All the patients enrolled in our study underwent conventional contrast enhanced MRI with a 1.5 T scanner (Magnetom Aera; Siemens Healthcare, Erlangen, Germany) with a phased-array body coil covering the upper abdomen prior to RFA. MRI sequences was composed of T1-weighted in- and opposed-phase spoiled 3-dimensional gradient-echo sequences and fat-suppressed T2-weighted two-dimensional turbo-spin-echo; diffusion-weighted imaging using a single-shot echo-planar imaging pulse sequence with b values = 0, 200, and 800 s/mm^2^ utilizing respiratory triggering. Three-dimensional T1-weighted volumetric interpolated breath-hold examination was obtained once before and 3 times after intravenous administration. Acquisitions were performed at 25, 85, and 180 seconds after injection gadopentetate dimeglumine (Magnevist; Bayer HealthCare, Berlin, Germany) at a rate of 3 mL/s and at a dose of 0.1 mmol/kg during the hepatic arterial, portal, and delayed phases, respectively. The injection was immediately followed by a 30-mL saline flush via a power injector (Spectris; Medrad, Pittsburgh, PA). All detailed parameters of each sequences are shown in Table 1.

**Table 1:** MRI sequences and parameters.

Parameters	Repetition time (msec)	Echo time (msec)	Section thickness (mm)	N Excitations	Field of view (mm^2^)	Bandwith (Hz/pixel)	Flip angle (degree)
T1-weighted Imaging	5.21	2.38	3	1	400 × 262	320	10
T2-weighted Imaging	2410	101	6	1	380 × 380	260	140
Diffusion weighted Imaging	5300	75	6	2	380 × 308	1698	90
Contrast-enhanced Imaging	160	2.22	5	1	380 × 285	300	70

### 2.3 RFA

Our RFA procedures were percutaneously performed by 1 of 3 radiologists who had 13, 11, and 9 years of clinical experience, respectively. RFA procedures and data assessment were based on Detailed methods of RFA are described in previous studies.^[9,10]^ All patients were treated when they were under IV conscious analgesic sedation. Commercially available internally cooled electrode systems with generators (Cool-tip RF System, Covidien, Mansfield, MA, USA; VIVA RFA System, STARmed, Goyang, Korea) were performed for all patients with HCCs in our cohort. Various electrodes including the single electrode with a 2 cm active tip, or with an adjustable active tip (Proteus RF Electrode, STARmed), or a cluster-electrode (Cool-tip, Covidien; Octopus, STARmed) were used according to tumor size and equipment availability. The energy deposition algorithm used was consistent with the introduced protocols. Our strategy was to include an ablative margin of at least 0.5 cm of normal hepatic parenchyma surrounding the tumor, as well as the entire tumor itself, with the exception of the perivascular portions and subcapsular. Our strategy of RFA was finished when the hyperechoic ablation range was large enough to cover the entire tumor and the expected ablative margin.

### 2.4 Tumor response and follow-up and outcome assessment

After RFA, all the patients were subjected to follow-up screening ultrasound, contrast-enhanced CT, MRI or PET-CT and serum AFP checkups performed every 3 months for the first two years, and then every 4–6 months thereafter. RFA response was assessed in the light of the modified Response Evaluation Criteria in Solid Tumors (mRECIST).^[11]^ The mRECIST evaluation was based on the sum of unidimensional measurements of arterially-enhancing tumors of pre-RFA MR and 1 month postoperative CT or MR. The tumor response was evaluated as follows: complete response (namely complete disappearance of any intra-tumoral enhancement), partial response (namely at least a 30% reduction compared to baseline); progressive disease (namely at least a 20% increase from baseline or appearance of new tumors or metastases); and stable disease (namely any response that did not qualify for inclusion in other categories). In the cohort, according to mRECIST assessment, there were 89 patients in complete response, and 16 patients in partial response.

The primary outcome of our study was LTP. The LTP was mainly used to describe the new tumor lesions at the margin of the postoperative ablation area. The LTP was precisely defined as a newly observed enhancing tumor foci in the arterial phase with the washout in the delayed phase at the surrounding of ablation areas after obtainment of successful therapy according to the guidelines.^[9]^ The follow-up time was defined as the interval between the first RFA and either the first LTP or the last visit to the outpatient clinic before May 30, 2017.

### 2.5 Imaging analysis

Two blinded and independent radiologists (with 5 and 23 years of experience in abdominal imaging, respectively) assessed the images on a picture archiving communication system workstation and all imaging measurements were performed on the workstation. The third observer (with 30 years of experience in abdominal radiology) was invited for an opinion in cases of inconsistency, and a majority decision was reached and served for further analysis.

#### 2.5.1 Qualitative image analysis

The analyzed MRI findings included the tumor number, tumor diameter, tumor margins (well-/ill-defined), location (right/left/bi lobar), signal intensity (SI) on T1-weighted, T2-weighted, and arterial, portal venous and delayed phase images (defined as hyperintense, hypointense, or isointense compared to the surrounding hepatic parenchyma), the presence of cirrhosis, the tumor texture, the presence of rim enhancement (defined as presence of ring-like areas of enhancement with central relatively hypointense areas in arterial phase), peritumoral enhancement (defined as grossly parenchymal enhancement outside of the tumor border in the arterial phase that becomes isointense with background liver parenchyma in the later dynamic phase images, regardless of shape)

#### 2.5.2 Quantitative image analysis

All quantitative data were independently performed by an experienced abdominal radiologist (with 9 years of experience in hepatic MR imaging interpretation). Data were measured twice, and the average values were used to minimize measurement error. The apparent diffusion coefficient (ADC) values were generated by a mono-exponential fit of the signal intensity using b = 0 and 800 s/mm^2^. ADC values were measured using regions of interests on the ADC maps and all regions of interests were manually drawn to include the largest portion of the tumor with no adjacent hepatic parenchyma and avoiding large vessels, and hemorrhage.

### 2.6 Development and validation of the nomogram

To build predictive nomograms based on various conventional MR characteristics, we used a multivariable logistic regression model to recognize the preoperative clinical and radiological factors and then combined the results to construct preliminary prediction models. Additionally, the utility of the preliminary MRI-based nomogram was validated by the calibration curve and the Harrell concordance index (C-index). The calibration curve was used to graphically depict predicted outcomes versus observed outcomes. The C-index was used to assess the predictive efficiency and accuracy of the preliminary nomogram.

### 2.7 Statistical analysis

Statistical analysis was conducted using SPSS software (version 22.0; IBM, Armonk, NY and R project version 3.5.0 (http://www.r-project.org/). Continuous variables were reported as means ± standard deviation and categorical variables were presented as numbers and percentages. Uni- and multi-variable analyses for the distribution of time to LTP were evaluated using the Cox proportional hazards model. The survival curve was assessed by using Kaplan–Meier analyses via the log-rank test. The predictive value of the factors for determining LTP was performed by receiver operating characteristic curve analysis. Cut-off values were chosen by maximizing the Youden index, and the sensitivity and specificity were also calculated. The interobserver agreement of the MRI qualitative parameters was assessed by the Cohen κ coefficient. The κ-values was defined as poor if κ<0.20, fair if κ=0.21–0.40, moderate if κ=0.41–0.60, good if κ=0.61–0.80 and excellent if κ=0.81–1.00, respectively.

A predictive nomogram based on the MRI findings was performed using the rms package in R project. The performance of the preliminary developed nomogram was assessed via the C-index and a calibration curve. All tests were 2-sides and *P* value <.05 was defined as statistically significance.

## 3 Results

### 3.1 Baseline characteristics

**Figure 2:**
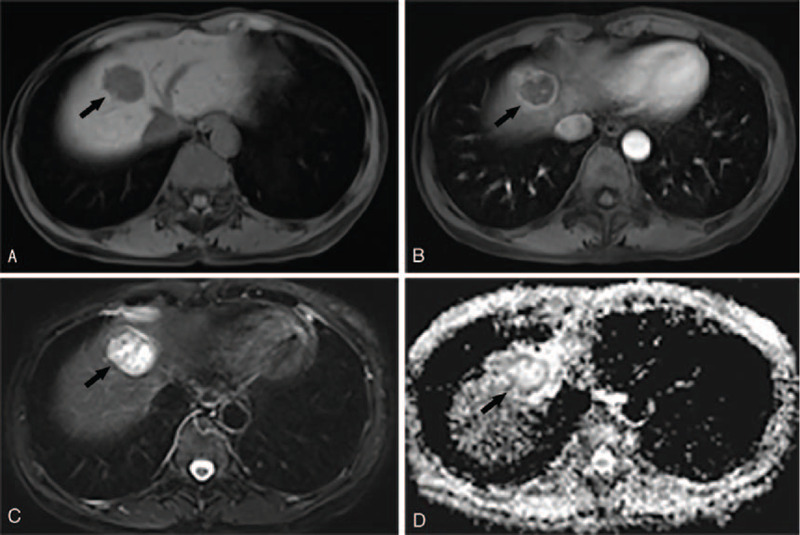
A patient in LTP-positive group after RFA. (A) T1-weighted imaging shows a well-defined hypointense tumor (black arrow). (B) MR imaging during the arterial phase the tumor exhibits rim enhancement (black arrow). (C) T2-weighted imaging shows a well-defined hyperintense tumor (black arrow). (D) On the ADC map, the tumor exhibits relatively hyperintense (black arrow).

This retrospective study finally enrolled 105 patients, 22 and 83 were in LTP-positive and LTP-negative groups, respectively. The mean follow-up periods were 364.9 days (median, 270 days; range, 31.0 - 1081.0 days). The clinical and radiological characteristics of all HCCs treated by RFA are showed in Table 2. In our cohort, 105 patients (median age, 58.2 ± 10.4 years; range, 29–82 years) with HCCs treated by RFA as a first-line therapy were finally included, containing LTP-positive (n = 22) and LTP-negative (n = 83). The average tumor size of LTP-negative/positive group was 1.8 ± 0.8 cm and 2.3 ± 1.1 cm, respectively. With regard to the clinical features in our cohort, AFP of patients in LTP-positive group was significantly larger than those in LTP-negative group (P < 0.001). However, all other clinical variables were not significantly different between both groups in the univariate Cox regression analysis (Table 3). Regarding the MRI characteristics of the HCCs treated by RFA, the tumors in LTP-positive group were more likely to show rim enhancement (*P* = .001), multiple nodules (*P* = .001), and lower ADC values (*P* < .001) compared to those in LTP-negative group (Fig. 2).

**Table 2:** The clinical and radiological parameters of the cohort.

Variables	LTP-negative (n = 83)	LTP-positive (n = 22)
Age (yr)^∗^	57.2 ± 8.8	58.4 ± 10.8
Sex		
female	17 (20.48)	7 (31.82)
male	66 (79.52)	15 (68.18)
Location		
left lobe	26 (31.33)	6 (27.27)
right lobe	54 (65.06)	15 (68.18)
bilobar	3 (3.61)	1 (4.55)
Hepatitis B virus		
presence	68 (81.93)	15 (68.18)
absence	15 (18.07)	7 (31.82)
Tumor size		
≤3 cm	65 (78.31)	13 (59.09)
>3 cm and ≤5 cm	18 (21.69)	9 (40.91)
Child-Pugh Class		
A	26 (31.33)	9 (40.91)
B	57 (68.67)	13 (59.09)
Tumor margin		
well-define	67 (80.72)	12 (54.55)
ill-define	16 (19.28)	10 (45.45)
Presence of liver cirrhosis	54 (65.06)	10 (45.45)
Pretreatment laboratory markers		
Albumin > 1.0mg/dL	22 (26.51)	7 (31.82)
AFP > 100 ng/mL	5 (6.02)	11 (50)
ALT > 40 U/L	29 (34.94)	10 (45.45)
AST > 40 U/L	45 (54.22)	14 (63.64)
GGT > 50 U/L	57 (68.67)	15 (68.18)
Tumor number		
1	75 (90.36)	14 (63.64)
2–3	8 (9.64)	8 (36.36)
Presence of rim enhancement		
presence	17 (20.48)	13 (59.09)
absence	66 (79.52)	9 (40.91)
Presence of peritumoral enhancement		
presence	11 (13.25)	4 (18.18)
absence	72 (86.75)	18 (81.82)
Signal in T2-weighted images		
hyperintense	68 (81.93)	16 (72.73)
isointense	13 (15.66)	5 (22.73)
hypointense	2 (2.41)	1 (4.55)
Signal on arterial phase		
hyperintense	72 (86.75)	17 (77.27)
isointense	10 (12.05)	4 (18.18)
hypointense	1 (1.20)	1 (4.55)
Signal on portal phase		
hyperintense	3 (3.61)	2 (9.09)
isointense	35 (42.17)	9 (40.91)
hypointense	45 (54.22)	11 (50)
Signal on delay phase		
hyperintense	2 (2.41)	1 (4.55)
isointense	32 (38.55)	8 (36.36)
hypointense	49 (59.04)	13 (59.09)
ADC (×10^–3^mm^2^/s)^∗^	1.09 ± 0.41	0.75 ± 0.26

**Table 3:** Uni- and multi-variate analyses of risk factors for LTP in HCCs treated by RFA.

	Univariate analysis			Multivariate analysis		
Risk factors	HR	95%CI	*P* value	HR	95%CI	*P* value
Age (y)	0.988	0.950, 1.028	.560			
Sex (male)	1.996	0.812, 4.908	.132			
Tumor margin (ill-define)	2.251	0.968, 5.234	.060			
Location						
left lobe	0.996	0.119, 8.361	.997			
right lobe	1.100	0.144, 8.422	.927			
bilobar^∗^	1					
Tumor size (≤3 cm)	0.240	0.519, 3.114	.600			
Child-Pugh Class (B)	1.351	0.576, 3.168	.488			
Presence of liver cirrhosis	0.544	0.235, 1.261	.156			
Pretreatment laboratory markers						
Albumin < 1.0mg/dL	1.165	0.475, 2.860	.739			
AFP > 100 ng/mL	6.722	2.563, 15.600	**<.001**	1.558	0.433, 5.610	.497
ALT < 50 U/L	1.259	0.543, 2.921	.591			
AST < 40 U/L	1.401	0.588, 3.343	.447			
GGT > 60 U/L	1.018	0.415, 2.499	.969			
Tumor number						
1^∗^	1					
2–3	4.698	1.961, 11.257	**.001**	1.847	0.703, 4.850	.213
Rim enhancement [present]	3.795	1.618, 8.902	**.002**	2.689	1.026, 7.049	**.044**
Presence of peritumoral enhancement [present]	1.059	0.357, 3.138	.918			
Signal in T2-weighted images						
hyperintense^∗^	1					
isointense	3.330	0.429, 25.829	.250			
hypointense	1.564	0.573, 4.273	.383			
Signal on arterial phase						
hyperintense^∗^	1					
isointense	1.978	0.262, 14.921	.508			
hypointense	1.294	0.434, 3.854	.644			
Signal on portal phase						
hyperintense^∗^	1					
isointense	0.403	0.086, 1.702	.203			
hypointense	0.372	0.086, 1.884	.248			
Signal on delay phase						
hyperintense	1					
isointense	0.581	0.075, 4.481	.602			
hypointense	0.607	0.075, 4.898	.604			
ADC (×10^−3^mm^2^/s)	0.066	0.017, 0.256	**<.001**^†^	0.136	0.020, 0.899	**.038**

### 3.2 Interobserver agreement for qualitative MRI characteristics

The observers showed excellent inter-observer agreement for the rim enhancement (κ = 0.884), presence of peritumoral enhancement (κ = 0.835), the ADC values (κ = 0.874) and SI on portal and delayed phase (κ = 0.895, 0.836, respectively) and good agreement for the tumor margin (κ = 0.734), SI in T2-weighted images (κ = 0.726), SI on arterial phase (κ = 0.794), and presence of liver cirrhosis (κ = 0.715).

### 3.3 Multivariate Cox regression analysis of the clinical and MRI variables associated with LTP

**Figure 3:**
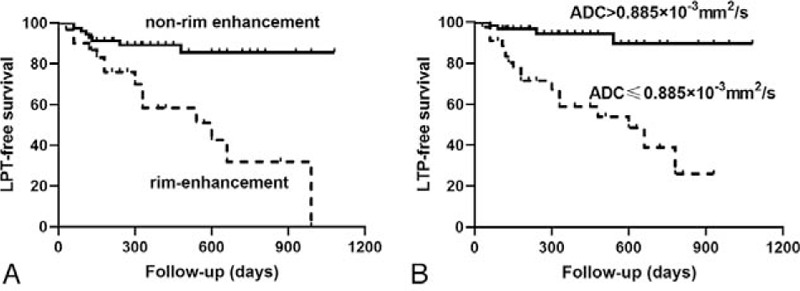
LTP-free survival of patients with HCCs treated by RFA. (A) Patients with rim enhancement vs non-rim enhancement. (B) high ADC values (> 0.885 × 10^−3^mm^2^/s) vs low ADC values (≤ 0.885 × 10^−3^mm^2^/s).

All significant clinical and imaging variables above were subsequently entered into multivariate Cox regression analysis. In multivariate analysis, MRI findings including ADC values (hazard ratio 0.066; 95% confidence interval, 0.020–0.899; *P* = .038) and rim enhancement (hazard ratio, 2.689; 95% confidence interval, 1.026–7.049; *P* = .044) were significant independent predictors of LTP after RFA (Table 3). In receiver operating characteristic analysis, the cut-off value of ADC was 0.885 × 10^–3^mm^2^/s for LTP prediction. The sensitivity, specificity, and area under the receiver operating characteristic curve of the rim enhancement and ADC value were 59.09%, 79.52%, 0.693 and 81.82%, 68.67%, 0.773, respectively. The 1-, 2-, and 3-year LTP-free survival in patients with rim enhancement were 73.3%, 63.3%, and 60%, respectively. The 1-, 2-, and 3-year LTP-free survival in patients with relatively low ADC values (< 0.885 × 10^–3^ mm^2^/s) were 68.2%, 61.4%, and 59.1%, respectively and these rates were significantly lower than those in their counterparts with non-rim enhancement and relatively high ADC values (Fig. 3)

### 3.4 Preliminary prediction MRI-based nomogram for LTP

**Figure 4:**
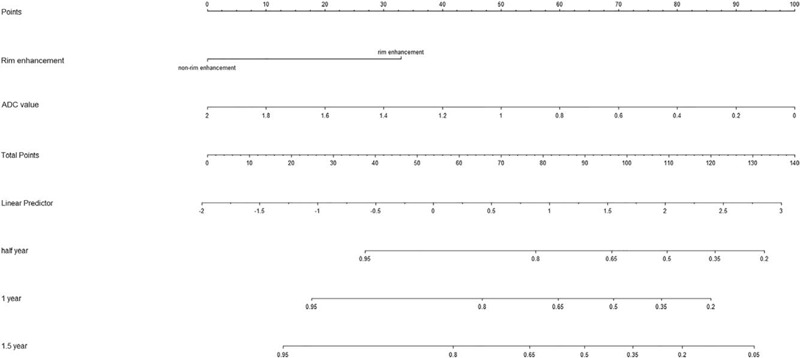
Nomogram was developed including 2 MRI features. The value for an individual patient with HCC treated by RFA is located on each variable axis and a line is drawn upwards to determine the number of points received for each variable value. The sum of values is located on the total points axis, and the line is drawn downwards to the survival axes to indicates the likelihood of 0.5-, 1-, 1.5-year LTP-free survival.

To create a more effective and accurate predictive model, we tried to integrate the statistically radiological prognostic indicators developed by multivariate analysis to create a preliminary predictive nomogram (Fig. 4). In our cohort, the C-index for LTP prediction with the developed nomogram was 0.790 (95% CI: 0.675–0.904) which is higher than that for rim enhancement and ADC values (Table 4). The higher C-index of the preliminary MRI-based nomogram is considered as the best performance was acquired when various MRI parameters were included.

**Table 4:** Discriminatory capabilities of developed nomogram and MRI independent variables.

	LTP	
Factors	C-index	95% CI
Rim enhancement	0.682	0.574, 0.792
ADC values	0.786	0.671, 0.901
Nomogram incorporating (rim enhancement + ADC value)	0.790	0.675, 0.904

### 3.5 Validation of the predictive accuracy of the MRI-based nomogram

**Figure 5:**
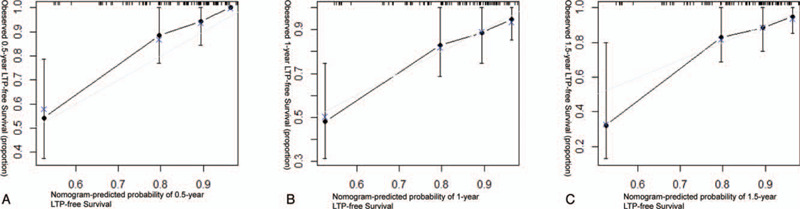
The calibration curves indicate LTP-free survival at (A) 0.5 years, (B) 1 years and (C) 1.5 years.

The calibration curves of our developed MRI-based nomogram graphically represented the bias-corrected line close to the most optimal curve (the 45-degree line), implying good consistency between prediction and observation at 0.5, 1, 1.5 years of HCCs treated by RFA as the first-line therapy (Fig. 5A–C).

## 4 Discussion

In our study, we demonstrated that rim enhancement and ADC value are independent predictive factors for LTP of HCC after RFA. In addition, we developed preliminary prediction model based on conventional MRI variables that enabled reliable prediction of LTP after curative RFA compared with the radiological finding alone.

Rim enhancement was more frequently observed in LTP-positive HCCs after RFA as the first-line therapy in our cohort. Rhee H, et al reported that enhancement pattern could be as usefully for predicting early recurrence of HCC after curative resection as pathologically determined microvascular invasion.^[12]^ Previous studies have been suggested that the rim enhancement pattern might indicate poor differentiation, rapid growth.^[13,14]^ Besides, several studies have also reported this enhancement pattern might be correlated to the absence of a capsule, infiltrative growth, and microvascular invasion as well as rapid progression with central necrosis.^[15,16]^ This may partially explain why patients with rim enhancement are more likely to LTP after RFA as first-line therapy.

Additionally, we also demonstrated the role of the ADC value in the preoperative prediction of LTP in patients with HCCs receiving RFA at the same time. Previously retrospective studies have shown the importance of ADC measurement for predicting response to surgical resection or transarterial chemoembolisation in patients with HCC.^[17–19]^ Mannelli et al reported that HCCs with lower ADC (b = 0, 50 and 500 s/mm^2^) in the pre-operate MR imaging are more likely have poor and incomplete response to transarterial chemoembolization.^[20]^ Lee, S et al have reported ADC value is a significant risk factor for early recurrence of single HCC tumors after surgical resection. Several prior studies have also evaluated the association between diffusion-weighted imaging and histological grade of HCC.^[21]^ As histological grade of HCC increases, cellular atypia of HCC, including mitotic activity and nucleus/cytoplasm ratio increases which might decrease diffusion of water molecules in the intracellular space and further lead to a reduced ADC value, likely partly explaining the results in our study.^[22]^

Preoperative imaging prediction of LTP of HCCs after RFA as the first-line treatment is of great importance because therapeutic outcome of RFA as the first-line treatment is more likely to be affected by microvascular invasion and vascular distribution around the tumor compared to curative resection.^[23]^ Hence, we presented a preliminary MRI-based nomogram created from multivariate Cox regression analysis as a tool for individualized risk evaluation. Compared to the complicated equation and calculations of an underlying logistic model, the developed nomogram enables clinician to graphically and easily compute the numerical probability of a clinical prognosis. In our study, to the best of our knowledge, we are the first to try to develop a new model that incorporates various MRI characteristics that can improve the predictive accuracy over that of MRI characteristics alone. In addition, further validation of the preliminarily developed model will be important to avoid overfitting of the model and demonstrate its usefulness and accuracy. Therefore, calibration curves were used to represent good consistency between nomogram-evaluated and actually observed probability and the C-index indicated that the MRI-based nomogram improves the accuracy of prediction.

There are several limitations in our study. First, there might have been inherent selection and verification biases because of the retrospective design. Second, although this study had a relatively large sample of LTP-positive of HCCs treated by RFA, our results may not represent the true spectrum of HCCs. Besides, the patients with HCCs treated by RFA as the first-line therapy were enrolled from a single hospital in China. The internal and external validation were not analyzed due to the limit of the sample size. Hence, large sample and multicenter prospective studies should be collected to further confirm our results and exclude the selective bias. Third, although the ablative factors of RFA was not incorporated in our analysis, we followed the fixed treatment protocol in terms of RFA throughout the study period. Fourth, the optimal cut-off ADC value used in our study may not be appropriate to all studies due to the differences in b-values and apparatus. Finally, despite of the feasibility and efficacy of this tool, this preliminary MRI-based nomogram model did not include all clinical variables. Further validations in other cohort are still needed to estimate the model's accuracy and feasibility.

Despite these limitations, to our knowledge, this is the first study evaluating the prognostic factors associated with RFA and conventional MRI and developing a new prediction model. Our study suggested that the MRI characteristics were useful imaging factors for predicting the LTP of HCC after RFA. Moreover, we tried to incorporate various MRI characteristics for improving the predictive accuracy than from single parameters of MRI. These results could be helpful to individually stratify the patients with HCCs who are potential candidates for RFA as the first-line therapy.

In conclusion, we indicated conventional MR features, including rim enhancement and ADC value, as easy-accessible and useful variables to predict the LTP of HCCs after RFA. Additionally, the preliminary MRI-based nomogram including various MRI parameters suggested a superior predictive model and may be helpful individually risk stratification of patients with HCC after RFA.

## Acknowledgments

We also thank Zhongjie Wang and Hong Ye from Interventional Diagnosis and Treatment Center, Zhoushan hospital of Zhejiang University for their technical assistance in patient surgery and image analysis.

## Author contributions

**Conceptualization:** Zhouchao Hu, Guoqiang Zhang.

**Data curation:** Nanna Yu, Jingang Yan.

**Formal analysis:** Nanna Yu.

**Funding acquisition:** Zhouchao Hu.

**Investigation:** Zhouchao Hu, Nanna Yu.

**Methodology:** Heping Wang, Jingang Yan.

**Project administration:** Heping Wang.

**Resources:** Heping Wang, Jingang Yan.

**Software:** Heping Wang, Jingang Yan.

**Supervision:** Zhouchao Hu, Shibo Li, Guoqiang Zhang.

**Validation:** Shibo Li, Jingang Yan, Guoqiang Zhang.

**Visualization:** Zhouchao Hu, Shibo Li, Guoqiang Zhang.

**Writing – original draft:** Zhouchao Hu, Guoqiang Zhang.

**Writing – review & editing:** Zhouchao Hu, Guoqiang Zhang.
